# Structural basis for dysregulation of aminolevulinic acid synthase in human disease

**DOI:** 10.1016/j.jbc.2022.101643

**Published:** 2022-01-28

**Authors:** Jessica L. Taylor, Breann L. Brown

**Affiliations:** 1 Department of Biochemistry, Vanderbilt University School of Medicine, Nashville, Tennessee, USA; 2 Center for Structural Biology, Vanderbilt University School of Medicine, Nashville, Tennessee, USA

**Keywords:** heme, aminolevulinic acid synthase, X-ray crystallography, enzyme structure, protein structure function, mitochondria, pyridoxal phosphate, ALA, aminolevulinic acid, ALAS, δ-aminolevulinic acid synthase, ClpX, Caseinolytic Mitochondrial Matrix Peptidase Chaperone Subunit X, PLP, pyridoxal phosphate, SCS, succinyl-CoA synthetase, TCA, tricarboxylic acid cycle, XLSA, X-linked sideroblastic anemia, XLP, X-linked protoporphyria

## Abstract

Heme is a critical biomolecule that is synthesized *in vivo* by several organisms such as plants, animals, and bacteria. Reflecting the importance of this molecule, defects in heme biosynthesis underlie several blood disorders in humans. Aminolevulinic acid synthase (ALAS) initiates heme biosynthesis in α-proteobacteria and nonplant eukaryotes. Debilitating and painful diseases such as X-linked sideroblastic anemia and X-linked protoporphyria can result from one of more than 91 genetic mutations in the human erythroid-specific enzyme ALAS2. This review will focus on recent structure-based insights into human ALAS2 function in health and how it dysfunctions in disease. We will also discuss how certain genetic mutations potentially result in disease-causing structural perturbations. Furthermore, we use thermodynamic and structural information to hypothesize how the mutations affect the human ALAS2 structure and categorize some of the unique human ALAS2 mutations that do not respond to typical treatments, that have paradoxical *in vitro* activity, or that are highly intolerable to changes. Finally, we will examine where future structure-based insights into the family of ALA synthases are needed to develop additional enzyme therapeutics.

The mitochondrion is a key hub for metabolic pathways, including fatty acid synthesis and the tricarboxylic acid (TCA) cycle. One critical metabolic process that occurs in the mitochondrion is heme biosynthesis. Heme is a cofactor necessary for nearly all organisms. Its functions range from carrying oxygen in hemoglobin, facilitating electron transport, detoxifying xenobiotics, and regulating transcription (1). Heme is an essential molecule, but free heme or heme precursors are highly toxic to the cell and aberrant cellular levels lead to multiple disorders (2, 3, 4).

Deciphering the mechanism of heme biosynthesis initiation will be critical to understanding the pathology of heme-related diseases. David Shemin first characterized the initial steps of heme biosynthesis that occur in α-proteobacteria, fungi, and mammals by himself ingesting ^15^N-glycine and analyzing his blood over time. Shemin *et al.* determined that the first heme precursor, aminolevulinic acid (ALA), was produced from the condensation of glycine and succinyl-CoA (Fig. 1) (5, 6, 7). Later studies using bacteria and chicken cell extracts identified that this reaction is catalyzed by the enzyme 5-aminolevulinic acid synthase (ALAS; EC 2.3.1.37) (8, 9, 10). This enzyme is the first and rate-limiting enzyme (excluding *Saccharomyces cerevisiae* and possibly other yeast and fungal species) initiating heme biosynthesis in α-proteobacteria and the mitochondria of several nonplant eukaryotes, and thus, it can be described as the gatekeeper of heme biosynthesis (11, 12).

**Figure 1:**
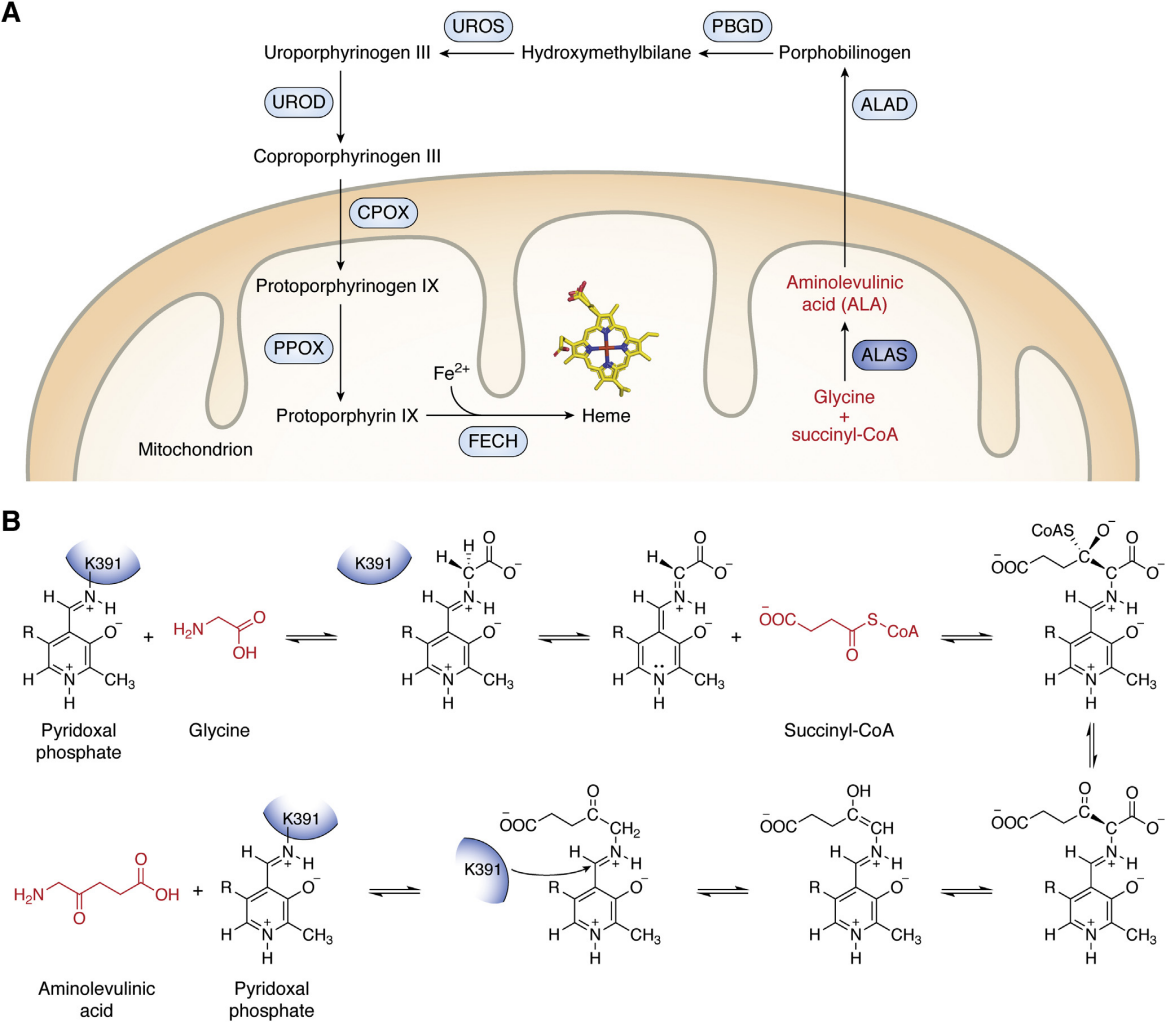
**Eukaryotic heme biosynthesis is initiated by aminolevulinic acid synthase.***A*, aminolevulinic acid synthase (*dark blue circle*) initiates heme biosynthesis in the mitochondrion of nonplant eukaryotes by catalyzing the condensation of glycine and succinyl-CoA to ALA (*red labels*). Other enzymes involved in the pathway are indicated in *light blue circles* whereas intermediates are labeled in *black*. *B*, reaction schematic for ALAS enzymes in which cofactor pyridoxal phosphate and active site lysine (K391) are used for the condensation reaction. Residue numbering is based on human ALAS2. ALA, aminolevulinic acid; ALAS, aminolevulinic acid synthase.

Aminolevulinic acid synthase belongs to the α-oxoamine synthase family of pyridoxal phosphate (PLP)-dependent enzymes that primarily catalyze condensations of amino acids and acyl-CoA thioesters (13, 14). These enzymes exist as homodimers with each PLP half-site buried at the dimer interface. Much of the work detailing the catalytic mechanism of ALAS was determined using murine ALAS2 (reviewed in refs (11, 15) and Fig. 1*B*). Pyridoxal phosphate is covalently bound to a catalytic lysine residue at the active site of the enzyme (the internal aldimine). Upon binding the first substrate glycine, the lysine is exchanged for the amino group of the substrate forming an external aldimine. Next, the second substrate succinyl-CoA binds and, after condensation, a quinonoid intermediate is formed. Protonation of this intermediate yields the ALA-external aldimine. The PLP cofactor provides an electron sink to stabilize the transient reaction intermediates, and the rate-limiting step of the reaction is the release of ALA product concomitant with a conformational change in ALAS (16, 17, 18). The ALA product is then transported out of the mitochondrion for the intermediate steps of heme biosynthesis, which ultimately concludes back inside the mitochondrial matrix where mature heme is made (Fig. 1*A*).

Although heme is produced in all cells, there are several differences between how heme biosynthesis is controlled in developing erythroid cells *versus* others (2). Vertebrates have two *ALAS* isoforms that are approximately 66% similar and 60% identical (19). *Aminolevulinic acid synthase 1*, located on chromosome 3, is a general housekeeping enzyme that is ubiquitously expressed (20). *Aminolevulinic acid synthase 2*, located on the X chromosome, is an erythroid-specific isoform responsible for initiating heme biosynthesis during erythropoiesis (21). In humans, over 85% of heme produced in the body is for red blood cell development (1). *Aminolevulinic acid synthase* *1* is negatively regulated by the intracellular concentration of heme both at the gene and protein level. Heme leads to the downregulation of *ALAS1* either by promoting gene repression through transcription factor binding to its heme regulatory element (22) or *via* downregulation of other positive transcription factors (23). At the protein level, ALAS1 is regulated by heme either by preventing mitochondrial import or by promoting protease degradation (24, 25). Conversely, *ALAS2* is positively regulated by different mechanisms including intracellular iron concentrations. The master transcription factor for erythroid development GATA1 binds canonical motifs within the *ALAS2* gene to activate transcription (26, 27, 28). The *ALAS2* gene promoter contains a noncanonical TATA motif that binds GATA1 and TATA-binding protein for maximal expression (29). In iron-rich conditions, the binding of iron-responsive proteins to the 5′ untranslated region of *ALAS2* is alleviated allowing for transcription (30). Post-transcriptionally, *ALAS2* is regulated by the long noncoding RNA Urothelial carcinoma-associated 1, which stabilizes *ALAS2* mRNA (31). To date, there is no experimental structure of human ALAS1. However, the recent crystal structure of human ALAS2 provides insight into how this gatekeeper functions for optimal heme production but also how mutations lead to various blood disorders (32).

Recent structural reports of ALAS from multiple organisms revealed key conserved ALAS features necessary for catalysis but raised questions about how critical catalytic regions control human ALAS2 activity. This review will focus on how various disease mutations may alter the ALAS2 structure. The structural determination of how disease-producing mutations alter the structural stability of an enzyme may lend critical information as to the mechanisms of individual mutations, giving the ability to develop targeted and personalized therapeutics.

## Aminolevulinic acid synthase structure and function

**Figure 2:**
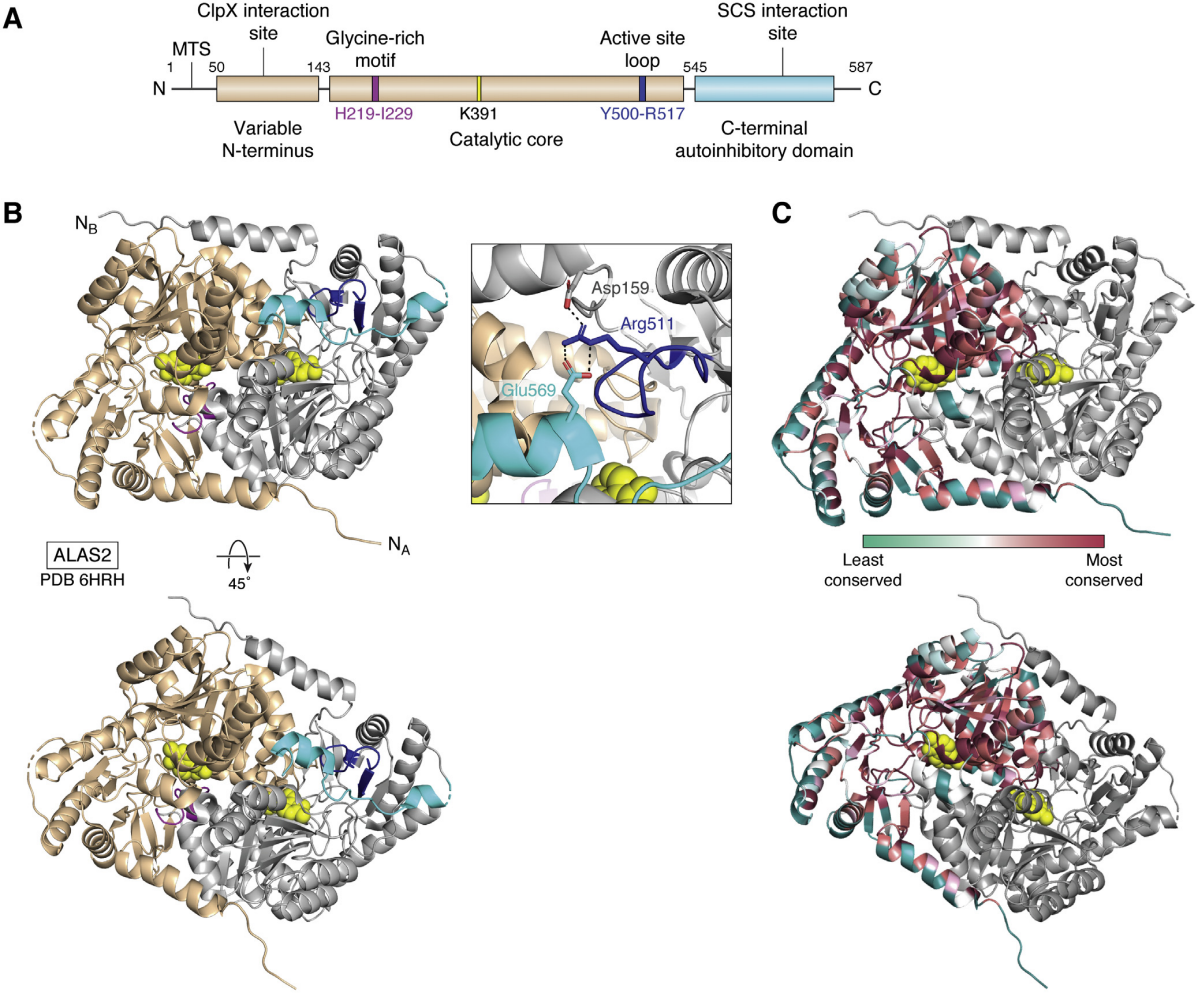
**Structure of human ALAS2.***A*, domain architecture of eukaryotic ALAS enzymes with catalytically important regions highlighted (MTS is the mitochondrial targeting sequence). Aminolevulinic acid synthase enzymes have a variable N-terminal domain that is believed to meditate ClpX recognition, a conserved catalytic core that contains the Glycine-rich motif (*magenta*), catalytic lysine (*yellow*), and active site loop (*dark blue*). There is a C-terminal, eukaryote-specific autoinhibitory domain (*cyan*) that was shown to interact with SCS. Residue numbers are indicated for human ALAS2. *B*, structure of human ALAS2 homodimer with one subunit in *tan* and the second subunit in *gray* (PDB ID 6HRH). Catalytically important regions are colored as in (*A*). The *bottom panel* is rotated 45 degrees about the indicated axis. *Inset*, residues Asp159, Arg511 of the active site loop, and Glu569 of the C-terminal extension form a salt bridge interaction network reported to play an autoinhibitory role in ALAS2 activity. *C*, sequence conservation of human ALAS2. The ConSurf server was used to identify ALAS homologs *via* PSI-BLAST (38). The conservation scores of this alignment are then mapped onto the structure of human ALAS2 as a color-coded heat map from variable (*turquoise*) to highly conserved (*maroon*) residues. ALAS, aminolevulinic acid synthase; ClpX, Caseinolytic Mitochondrial Matrix Peptidase Chaperone Subunit X; SCS, succinyl-CoA synthetase.

Human ALAS2 exists as a homodimer with each protomer comprising an N-terminal region (amino acids 1–142) that harbors the mitochondrial targeting sequence, a conserved catalytic core (amino acids 143–544) containing the PLP cofactor and active site, and a C-terminal extension (amino acids 545–587) that is a primary source of sequence variability among organisms and is only present in eukaryotes (Fig. 2) (32). The C-terminal extension of vertebrate ALAS2 plays an autoinhibitory role in catalysis (33) and is also the region where all X-linked protoporphyria (XLP) mutations reside, an inherited disease that results from enzyme hyperactivity (see below). The first reported crystal structures of ALAS enzymes were from *Rhodobacter capsulatus*, which is 49% identical to human ALAS2 (16). These structures of *R. capsulatus* ALAS in the presence of the PLP cofactor, glycine, or succinyl-CoA revealed that ALAS adopts ‘open’ and ‘closed’ conformations. These widen the active site in the absence of substrate and narrow the active site in the presence of substrates.

There are several key-conserved ALAS structural elements (Fig. 2*A*). Kardon *et al.* reported the N-terminal segment in yeast ALAS interacts with Caseinolytic Mitochondrial Matrix Peptidase Chaperone Subunit X (ClpX), an ATP-dependent protein quality control enzyme, to facilitate PLP cofactor binding (34, 35). Next, the conserved glycine-rich motif (amino acids His219-Ile229 in human ALAS2) helps to mediate cofactor and substrate binding (36). This region is flexible in the absence of a bound cofactor (37). Importantly, multiple residues in this loop, including a conserved arginine (Arg227 in human ALAS2) that does not interact directly with the bound PLP cofactor, cannot tolerate mutation (16). Arg227 mediates a salt bridge with Glu240 in human ALAS2, an interaction that is conserved in yeast ALAS, presumably restricting the dynamics of this loop and capping the substrate-binding pocket. There is a conserved catalytic lysine residue at the active site that forms a covalent Schiff base with the PLP cofactor to activate the enzyme for catalysis (Fig. 1*B*). The ALAS active site loop (residues Tyr500-Arg517 in human ALAS2) was shown to provide the regulatory gate for ALA product release. The active site loop interacts with the eukaryote-specific C-terminal extension. In addition to these conserved regulatory regions, several sites are not conserved in ALAS enzymes (Fig. 2*C*) (38). These lesser-conserved patches are typically surface exposed. How these regions control enzyme activity, potentially by mediating protein–protein or protein–ligand interactions, remains to be determined.

The first reported eukaryotic ALAS structure from *S. cerevisiae* provided structural insights into how the PLP cofactor stabilizes the active site and positions catalytic residues for binding both glycine and succinyl-CoA substrates (37). The ALAS active site accommodates glycine as the first substrate and succinyl-CoA as the second, which are condensed to yield ALA (Fig. 1*B*). This reaction is initiated by binding a PLP cofactor, the active form of Vitamin B6, which is required for ALAS activity. The *S. cerevisiae* C-terminal extension is dissimilar from the C-terminal region of metazoan ALAS2 in both sequence and structure. Intriguingly, deletion of this region decreases enzymatic activity in *S. cerevisiae* ALAS (37), whereas deletion of the C-terminus of human ALAS2 results in increased enzymatic activity; the latter underlies XLP (39). The human ALAS2 structure revealed that the C-terminal extension both impacts ALA release during catalysis and restricts movement of the active site loop through a salt bridge network between residues Asp159 of the catalytic core, Arg511 of the active site loop, and Glu569 of the C-terminal extension (Fig. 2*B*) (32). This salt bridge network must be disrupted to access the catalytic residues for both substrate binding and product release, imposing an autoinhibitory function of the C-terminal extension. Thus, the structural position of the human ALAS2 C-terminal extension provides insight into how this peptide regulates overall enzyme activity.

## Aminolevulinic acid synthase 2 and disease

**Figure 3:**
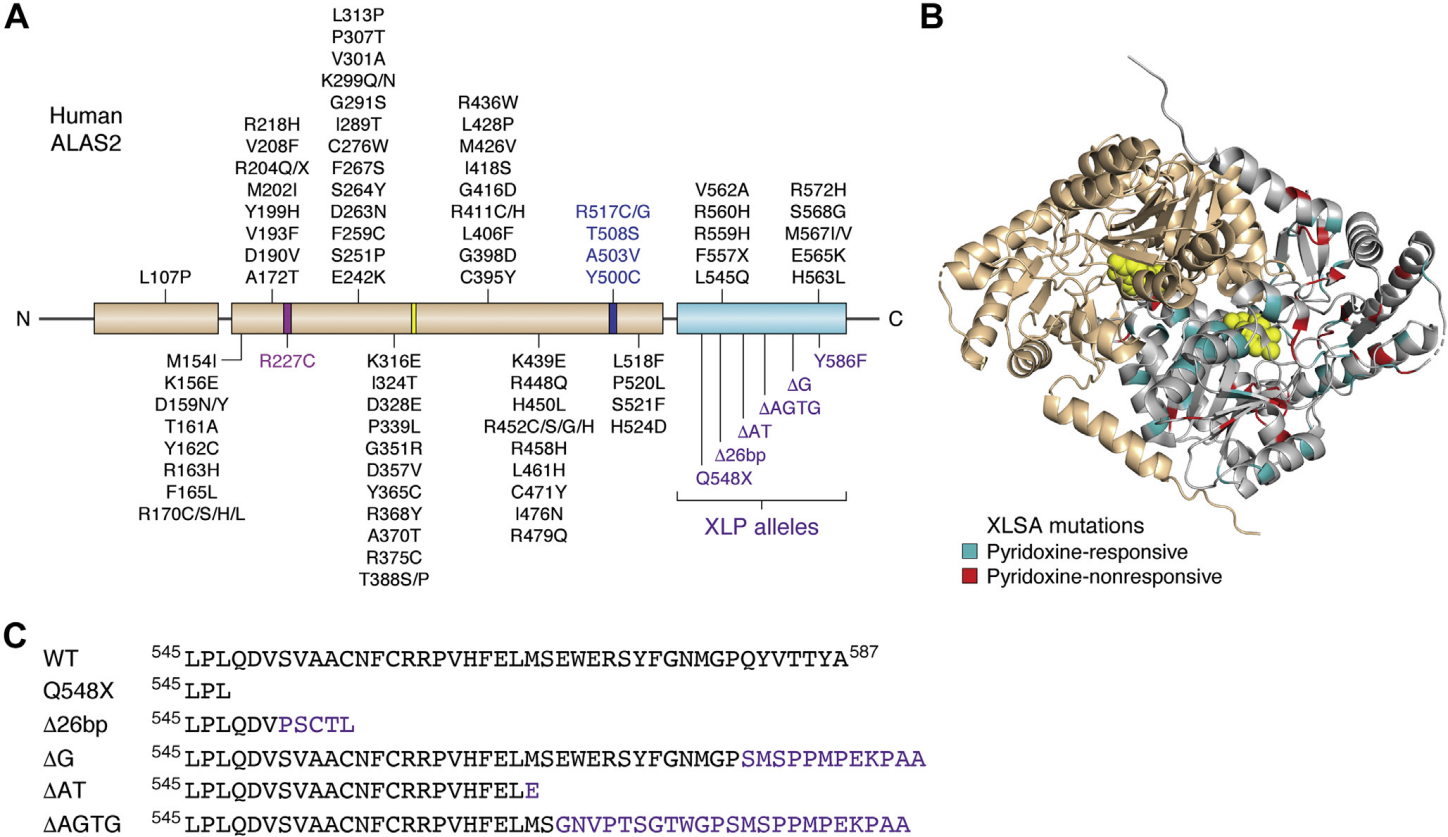
**Structural correlation of ALAS2 disease mutations.***A*, aminolevulinic acid synthase domain architecture (as in Fig. 2*A*) with each of the currently identified disease alleles labeled. X-linked sideroblastic anemia mutations are shown in *black* except for mutations in catalytically important regions, which are colored as in Figure 2*A*. All XLP alleles reside in the C-terminal autoinhibitory domain and are listed in *purple*. *B*, all reported XLSA mutations are mapped onto one protomer of the structure of human ALAS2, with pyridoxine-responsive mutations colored *teal* and pyridoxine-nonresponsive mutations colored *red*. *C*, sequence of XLP deletion and frameshift mutations affecting the ALAS2 C-terminal extension. Wild-type ALAS2 sequence is shown on the top line. ALAS, aminolevulinic acid synthase; XLP, X-linked protoporphyria; XLSA, X-linked sideroblastic anemia.

More than 90 disease-associated variants have been reported in the *ALAS2* gene (Fig. 3*A*). Clinical mutations in *ALAS2* are mainly missense mutations, which make up over 85% of all *ALAS2* mutations to date (40). Other mutations, which manifest as insertions or deletions, are present in the promoter region of the *ALAS2* gene or lead to alternative splice variants (40). The ALAS2 amino acid mutations that cause X-linked sideroblastic anemia (XLSA, OMIM 300751) are widely dispersed throughout the structure rather than being localized in one region (Fig. 3*B*). X-linked sideroblastic anemia is characterized by cellular iron overload and reduced heme (and hemoglobin) production. Certain XLSA mutations lead to a decrease in ALAS2 activity. Symptoms of this disease range from fatigue, dizziness, and rapid heartbeat to heart disease and cirrhosis (41). X-linked sideroblastic anemia is most notably recognized by ring sideroblasts in the bone marrow. X-linked sideroblastic anemia is the most common form of congenital sideroblastic anemia and can manifest anytime from infancy to late adulthood (42). Successful treatments for XLSA include phlebotomy, chelation, pyridoxine supplementation, and recently, gene editing (43, 44). Pyridoxine supplementation is a prevalent treatment for XLSA as it is metabolized in the liver to the active form of PLP (45), and of the 91 XLSA mutations, 53 have historically responded to pyridoxine treatment in one or more patients (Table 1). The responsiveness to pyridoxine has commonly been attributed to the point mutations altering an amino acid located in the vicinity of the PLP-binding site (46, 47, 48). However, there does not appear to be a relationship between pyridoxine responsiveness and the 3-dimensional location of a particular mutation (Table 1 and Fig. 3*B*).

**Table 1:** Known human ALAS2 XLSA mutations with pyridoxine response, structural location, and instability rank

Residue	Mutation	Pyridoxine responsive^a^	Location	Structural instability rank^b^	Reference
Leu107	Pro	No	N-terminus	N/A	(73)
Met154	Ile	No	N-terminus	Mild	(16)
Lys156	Glu	Yes	N-terminus	Mild	(74)
Asp159	Asn	Yes	N-terminus	Mild	(16)
Asp159	Tyr	Yes	N-terminus	Moderate	(16)
Thr161	Ala	No	N-terminus	Moderate	(73)
Tyr162	Cys	No	N-terminus	Moderate	(75)
Arg163	His	No	N-terminus	Moderate	(76)
Phe165	Leu	Yes	N-terminus	Mild	(16)
Arg170	Cys	Yes	N-terminus	Moderate	(77)
Arg170	Ser	Yes	N-terminus	Moderate	(16)
Arg170	His	Yes	N-terminus	Moderate	(16)
Arg170	Leu	Yes	N-terminus	Mild	(16)
Ala172	Thr	Yes	N-terminus	Moderate	(16)
Asp190	Val	No	N-terminus	Mild	(78)
Val193	Phe	Yes	Catalytic core	Moderate	(79)
Tyr199	His	Yes	Catalytic core	Moderate	(16)
Met202	Ile	Yes	Catalytic core	Moderate	(79)
Arg204	Gln	Yes	Catalytic core	Mild	(16)
Arg204	Truncation	No	Catalytic core	N/A	(16)
Val208	Phe	No	Catalytic core	Moderate	(80)
Arg218	His	No	Catalytic core	Mild	(56)
Arg227	Cys	No	Glycine-rich motif	Moderate	(81)
Glu242	Lys	Yes	Catalytic core	Moderate	(56)
Ser251	Pro	No	Catalytic core	Moderate	(82)
Phe259	Cys	Yes	PLP-interacting	Moderate	(79)
Asp263	Asn	Yes	Catalytic core	Moderate	(16)
Ser264	Tyr	Yes	Catalytic core	Unaffected	(83)
Phe267	Ser	Yes	Catalytic core	Mild	(79)
Cys276	Trp	No	Catalytic core	Moderate	(82)
Ile289	Thr	Yes	Catalytic core	Moderate	(84)
Gly291	Ser	Yes	Catalytic core	Moderate	(16)
Lys299	Gln	Yes	Catalytic core	Moderate	(16)
Lys299	Asn	No	Catalytic core	Moderate	(85)
Val301	Ala	No	Catalytic core	Moderate	(77)
Pro307	Thr	Yes	Catalytic core	Moderate	(79)
Leu313	Pro	No	Catalytic core	Severe	(86)
Lys316	Glu	Yes	Catalytic core	Mild	(79)
Ile324	Thr	No	Catalytic core	Severe	(75)
Asp328	Glu	No	Catalytic core	Moderate	(87)
Pro339	Leu	Yes	Catalytic core	Mild	(56)
Gly351	Arg	Yes	Catalytic core	Moderate	(16)
Asp357	Val	No	PLP-interacting	Moderate	(79)
Tyr365	Cys	No	Catalytic core	Moderate	(88)
Arg368	Tyr	No	Catalytic core	Mild	(87)
Ala370	Thr	No	Catalytic core	Mild	(89)
Arg375	Cys	No	Catalytic core	Moderate	(56)
Thr388	Ser	Yes	PLP-interacting	Mild	(16)
Thr388	Pro	Yes	PLP-interacting	Severe	(79)
Cys395	Tyr	Yes	Catalytic core	Moderate	(16)
Gly 398	Asp	No	Catalytic core	Severe	(82)
Leu406	Phe	Yes	Catalytic core	Mild	(48)
Arg411	Cys	Yes	Catalytic core	Moderate	(16)
Arg411	His	Yes	Catalytic core	Moderate	(16)
Gly416	Asp	Yes	Catalytic core	Moderate	(16)
Ile418	Ser	Yes	Catalytic core	Moderate	(90)
Met426	Val	Yes	Catalytic core	Mild	(16)
Leu428	Pro	Yes	Catalytic core	Severe	(91)
Arg436	Trp	No	Catalytic core	Mild	(92)
Lys439	Glu	Yes	Catalytic core	Mild	(47)
Arg448	Gln	Yes	Catalytic core	Mild	(16)
His450	Leu	Yes	Catalytic core	Unaffected	(79)
Arg452	Cys	Yes	Catalytic core	Mild	(79)
Arg452	Ser	Yes	Catalytic core	Mild	(16)
Arg452	Gly	Yes	Catalytic core	Mild	(56)
Arg452	His	Yes	Catalytic core	Mild	(16)
Arg458	His	No	Catalytic core	Mild	(93)
Leu461	His	Yes	Catalytic core	Severe	(94)
Cys471	Tyr	Yes	Catalytic core	Mild	(95)
Ile476	Asn	Yes	Catalytic core	Severe	(16)
Arg479	Gln	No	Catalytic core	Moderate	(96)
Tyr500	Cys	Yes	Active-site loop	Severe	(97)
Ala503	Val	No	Active-site loop	Moderate	(98)
Thr508	Ser	No	Active-site loop	Mild	(82)
Arg517	Cys	No	Active-site loop	Moderate	(82)
Arg517	Gly	No	Active-site loop	Moderate	(77)
Leu518	Phe	Yes	Catalytic core	Unaffected	(91)
Pro520	Leu	No	Catalytic core	Moderate	(77)
Ser521	Phe	No	Catalytic core	Mild	(87)
His524	Asp	Yes	Catalytic core	Moderate	(99)
Leu545	Gln	Yes	C-term extension	Severe	(79)
Phe557	Truncation	No	C-term extension	N/A	(54)
Arg559	His	No	C-term extension	Moderate	(93)
Arg560	His	Yes	C-term extension	Moderate	(16)
Val562	Ala	No	C-term extension	Moderate	(82)
His563	Leu	Yes	C-term extension	Mild	(79)
Glu565	Lys	Yes	C-term extension	Mild	(79)
Met567	Ile	No	C-term extension	Moderate	(54)
Met567	Val	No	C-term extension	Moderate	(54)
Ser568	Gly	No	C-term extension	Moderate	(54)
Arg572	His	Yes	C-term extension	Moderate	(56)

a Pyridoxine responsiveness is determined from published, clinical patient data according to the indicated reference.

b Instability ranks are classified as mild (ΔΔG > −1), moderate (−1 ≥ ΔΔG ≥ −3), or severe (ΔΔG < −3) as calculated using DeepDDG server (53).

The residues that interact directly with PLP cofactor in the human ALAS2 structure are Ser257, Cys258, Phe259, His285, Asp357, Val359, His360, Thr388, and Lys391 in Subunit A, and Thr420 and Thr421 in Subunit B (32). Three of these residues (Phe259, Asp357, and Thr388) are known to be mutated leading to XLSA. Interestingly, the Asp357Val variant has not yet been reported to respond to pyridoxine whereas the others do respond to pyridoxine treatment. Asp357 is a conserved residue that is responsible for providing specificity of cofactor binding (16, 32, 37), helps in the formation of a quinonoid reaction intermediate, and mutations in this residue are known to decrease PLP affinity in murine ALAS2 (49). Given its role in PLP binding, it is surprising that the Asp357Val variant does not respond to pyridoxine treatment. Further, only 8% of the total reported XLSA pyridoxine-responsive mutations affect the PLP-interacting residues (Table 1). The XLSA mutations that respond to pyridoxine supplementation encompass residues beyond those that directly interact with PLP. In fact, when the XLSA mutations are mapped to the ALAS2 structure, the pyridoxine-responsive mutations are scattered throughout the conserved catalytic core and the less-conserved C-terminal extension (Fig. 3*B*). This suggests there may be other ways that pyridoxine supplementation is an effective treatment for XLSA. Different mutations may cause structural perturbations that allosterically alter PLP affinity, affect ALAS2 dimerization, decrease enzyme stability and expression, and change substrate cooperativity and affinity. Pyridoxine supplementation may alleviate these structural and enzymatic effects through various mechanisms. Future work is necessary to uncover the specific mechanism for each individual mutation.

In addition to mutations causing XLSA, other mutations in the C-terminal extension of ALAS2 are the cause of XLP (OMIM 300752). X-linked protoporphyria results in a gain-of-function activity of ALAS2 (50). This causes a build-up of toxic heme porphyrin intermediates. Patients suffering from this disease may experience cutaneous symptoms such as swelling, scarring, and painful skin photosensitivity to more severe symptoms like enlargement of the spleen and chronic kidney disease (51). Importantly, all known XLP disease alleles affect the ALAS2 eukaryote-specific C-terminal extension (Fig. 3*C*) (50, 52). The recent crystal structure of ALAS2 revealed structural insights into how C-terminal XLP mutations result in increased ALAS2 activity. Namely, any deletion, replacement, or elongation of its C-terminal extension could interfere with the molecular interactions that stabilize its autoinhibitory role (Fig. 2*B*) (32). What has yet to be explained is how the dispersion and diverse nature of XLSA mutations result in the common phenotype of decreased heme production. We have classified the following XLSA mutations into three groups based on recorded properties of the disease in patients and properties of the mutant ALAS2 proteins. Many variants of the *ALAS2* gene are not responsive to treatment (Table 1). We hope to provide new ways to think about XLSA mutant proteins and future treatment based on structural and thermodynamic insights.

### Mutations found to be nonresponsive to pyridoxine treatment

Currently, 38 of the 91 total known XLSA mutations are not reported to respond to pyridoxine supplementation (Table 1). Mapping the 36 missense mutations onto the ALAS2 structure and comparing evolutionary conservation shows that, exclusive of the five mutations in the C-terminal extension, 22 of the 31 residues localize to an evolutionarily conserved portion of the structure. We analyzed ALAS2 mutations using the DeepDDG server (53) to calculate ΔΔG values for each mutation and then ranked by structural instability or defect (Table 1). Leu313Pro, Ile324Thr, and Gly398Asp were the three highest-ranked mutations predicted to result in severe protein folding destabilization. These nonpyridoxine responsive mutations are both surface exposed and buried, thus, their mechanism for destabilizing ALAS2 may vary.

Although specific information on every nonpyridoxine responsive mutant’s ability to bind PLP is not known, some of these variants have been purified and assessed for *in vitro* enzymatic activity (*e.g.*, D190V, R559H, M567V, and S568G) (44, 54, 55). The nonpyridoxine responsive mutants, just like the pyridoxine-responsive mutants, have variable activity. Some have decreased activity, whereas some have similar activity to WT, which indicates that at least a subset is able to competently bind the PLP cofactor (44, 54, 55). It is not known whether all of the nonpyridoxine responsive mutants are able to bind PLP. Purifying and testing for the presence of enzymatic activity can determine if these mutants are able to bind PLP.

Finally, several of the nonpyridoxine responsive mutations located outside of the C-terminal extension were often mutated to a dissimilar amino acid. One example is Tyr162Cys that changes from an aromatic, hydrophobic residue to a smaller, sulfur-containing residue. Ser251Pro is another example in which the residue changes from a polar residue to a cyclic, rigid residue. Finally, Arg436Trp converts a positively charged, basic residue to an aromatic, hydrophobic residue. Whereas in the C-terminal extension, the mutations were almost always (four out of five) mutated to a similar amino acid rather than involving a reversal of charge, size, or polarity (*i.e.*, hydrophobic to hydrophobic or positive and basic to positive and basic). It is not clear why certain XLSA mutants, even when the mutations are outside of the PLP-binding region, respond to pyridoxine and some do not. This information can be used in the development of alternative treatment options outside of pyridoxine supplementation, such as increasing protein expression or stabilizing the mutant proteins.

### X-linked sideroblastic anemia-causing mutations with normal ALAS2 enzymatic activity

XLSA can frequently be attributed to decreased ALAS2 activity *in vivo*. The decrease in enzymatic activity leads to a toxic buildup of iron in erythroblasts. There are multiple ALAS2 variants that maintain normal enzymatic activity *in vitro* when mutated to their corresponding XLSA mutation. There are at least seven known mutants that are reported to maintain normal enzyme activity (54, 56). These are Arg170His, Arg218His, Arg452His, Arg452Cys, Pro520Leu, Met567Val, and Arg572His. These occur in the catalytic core (R170H, R218H, R452H, R452C, and P520L) and in the C-terminal extension (M567V and R572H). We inquired if any of these mutations were in conserved regions using the ConSurf server, which uses multiple sequence alignments to analyze the evolutionary conservation of amino acids to highlight the regions of a protein that may be important for structure and function (38). Mutations Arg218His, Arg452His, Arg452Cys, Met567Val, and Arg572His exist in nonevolutionarily conserved residues according to ConSurf analysis (Fig. 2*C*), and these mutations were predicted to be among the least destabilizing (Table 1). In this group, four of the five arginine residues are mutated to a histidine, which is also a positively charged, basic amino acid. If the phenotype of WT enzymatic activity that is observed *in vitro* is also present in the corresponding XLSA patients, then these mutations must be decreasing heme production through means other than impacting ALAS2 activity. This could include disruption of an unidentified protein–protein interaction, a decrease in *ALAS2* expression, or an increase in ALAS2 turnover. Targeted treatment for these specific variants should include consideration of other molecular defects that arise from the mutations.

### Multivalent XLSA mutations

Finally, multiple amino acids are intolerant to changes, so-called multivalent mutations, which are a unique group of XLSA variants with variable pyridoxine responsiveness. These are the specific ALAS2 residues that are documented to be mutated to more than one other amino acid causing XLSA. Of the 91 XLSA mutations, only eight are known to be mutated to multiple residues. These are Asp159, Arg170, Lys299, Thr388, Arg411, Arg452, Arg517, and Met567. Generally, these types of amino acids are not particularly more susceptible to mutations compared to other amino acids (57). Of note, Thr388 is a specific PLP-interacting residue. When looking at each residue in the ALAS2 structure, five of the eight multivalent residues are surface exposed, with Arg170, Thr388, and Arg517 as exceptions. Intriguingly, the ΔΔG predictions for each multivalent mutation do not indicate severe folding defects. Within the multivalent mutations, residues Arg170, Arg411, and Arg452 are mutated to four other amino acids, and Asp159, Lys299, Thr388, Arg517, and Met567 are mutated to two other amino acids (Table 1). Many of these residues are predicted to be completely intolerant to mutation according to SIFT analysis, a web-based server that predicts the phenotypic effects of amino acid mutations based on sequence homology and the physical properties of a particular amino acid (58). Other than spatial availability, there may be selected pressure on these amino acids to be mutated. Thus, a detailed functional and structural analysis of these mutations may reveal new insight into the conserved role of these residues in all ALAS enzymes.

## Mutations yielding ALAS2 hyperactivity

**Figure 4:**
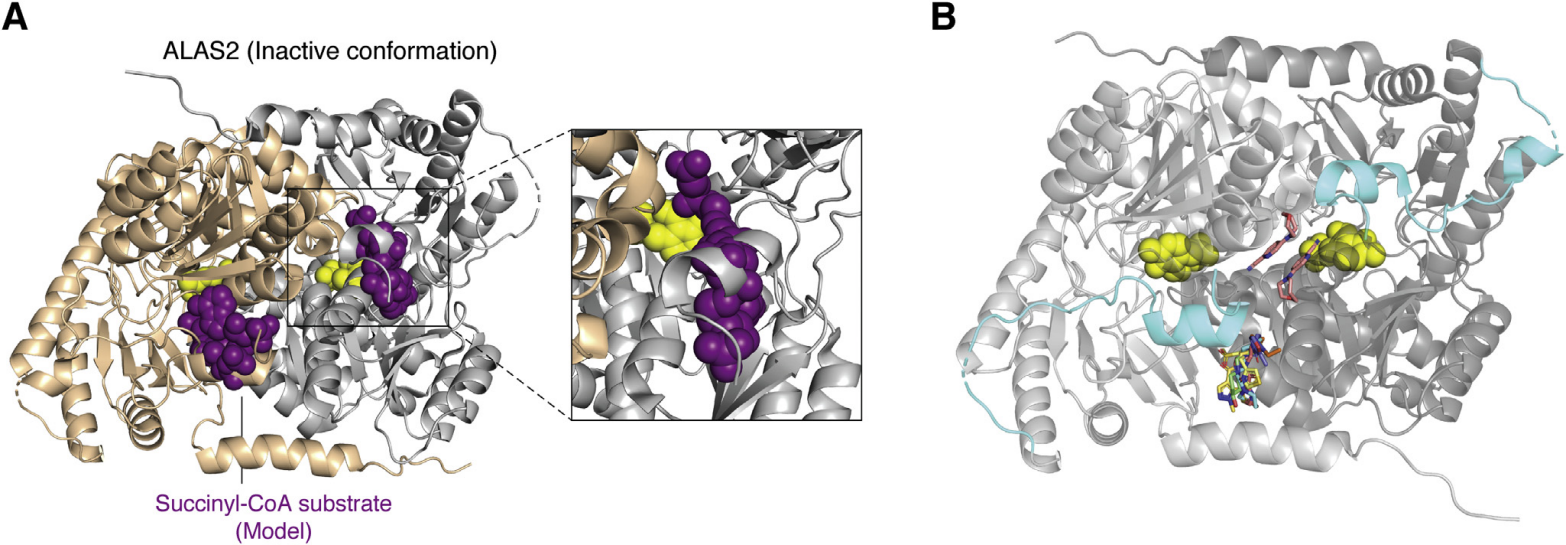
**ALAS2 structural insight for activation and therapeutic development.***A*, the current ALAS2 crystal structure was captured in the inactive conformation with the substrate-binding site occluded by a region of the C-terminal extension (helix α15). The succinyl-CoA substrate (*purple spheres*) was modeled into human ALAS2 using the homologous substrate-bound ALAS structure from *Rhodobacter capsulatus* (16). A close-up view of one active site (*inset*) shows that the C-terminal extension directly clashes with the putative succinyl-CoA substrate-binding pocket. *B*, fragment screening crystallization identified nine candidate compounds (*stick* representation) that bind in regions near the C-terminal extension (*cyan*). The following PDB files were superimposed onto the human ALAS2 structure: 5QQY, 5QR1, 5QRA, 5QRC, 5QRD, 5QQW, 5QQX, 5QRE, and 5QQU. ALAS, aminolevulinic acid synthase.

As opposed to the loss-of-function XLSA alleles, all the gain-of-function ALAS2 mutations involve truncations or frameshifts that alter the eukaryote-specific C-terminal extension (Fig. 3*C*). This region wraps around the outer surface of the catalytic core and meets together at the dimer interface. Importantly, the current ALAS2 structure was captured in an inactive conformation with the succinyl-CoA substrate-binding pocket occluded by a small, two-turn α helix spanning residues 568 to 575 (helix α15, Fig. 4*A*). Molecular dynamics simulations suggested that multiple salt bridge interactions must be broken for the C-terminal extension to reorient allowing for substrate binding (32). Although the last nine ALAS2 residues are disordered in the crystal structure, secondary and tertiary structure predictions indicate the presence of a β strand in that region (59, 60). The most structurally and functionally severe C-terminal XLP mutation, Gln548X, results in a truncation of the entire C-terminal extension (39, 52, 61). Two other XLP mutations resulting from frameshift deletions, either by deleting the last 20 amino acids (ΔAT) or by altering the last 19 amino acids and including a nonnative four-reside extension (ΔAGTG), ablate this α helix (50). The loss of this inhibitory interaction is believed to underlie the ALAS2 hyperactivity seen in XLP. The ΔG frameshift mutation substitutes seven residues after Pro580 and then elongates by an extra five residues (61). This frameshift is predicted to disrupt the final predicted β strand (59). The last XLP mutation, Tyr586Phe affects the penultimate residue in ALAS2 (62). Because this region is disordered in the ALAS2 crystal structure, it remains to be seen how this mutation modulates the position of the C-terminal region.

## Aminolevulinic acid synthase 2 and protein effectors

One outstanding question in the field is how do ALAS2 protein effectors modulate structure and function? The N-terminal region of yeast ALAS was reported to bind the ClpX unfoldase to accelerate cofactor binding and enzyme activity (34, 35). Recent studies show the role of the ClpX interaction in vertebrate ALAS2 may play a different function and lead to protein degradation (63). Regardless of its role, the interaction of ALAS with ClpX will undoubtedly uncover new conformational changes underlying enzyme regulation not captured in the current crystal structure. Aminolevulinic acid synthase was also shown to bind the TCA cycle enzyme succinyl-CoA synthetase (SCS); mutations in ALAS2 preventing interaction with SCS are associated with XLSA (54, 64). This interaction was believed to supply succinyl-CoA substrate to ALAS2 for heme biosynthesis. However, a different mitochondrial matrix TCA cycle enzyme, the E1 subunit of α-ketoglutarate dehydrogenase, was shown to bind ALAS2 in mouse erythroleukemia cells for this purpose (65). α-ketoglutarate dehydrogenase catalyzes the oxidative decarboxylation of α-ketoglutarate to succinyl-CoA (66, 67). This report raises important questions. Why does ALAS2 bind two TCA cycle enzymes that both produce succinyl-CoA? Is the binding of SCS and α-ketoglutarate dehydrogenase mutually exclusive or does a ternary complex exist? Are there different cellular conditions under which the binding of one partner is favored over the other? Finally, ALAS2 may reside in a larger, multi-protein assembly involving several other mitochondrial heme biosynthetic proteins (68). The purpose of this complex may be to shuttle substrates among enzymes and provide feedback regulation of ALAS2. It will be illuminating to uncover the structural identity of such a complex.

## Potential impact and future structural insight

The structures of ALAS enzymes from bacteria, yeast, and now humans highlight conserved and disparate features that may be amenable to structure-based therapeutic development. Bailey *et al.* (32) established a druggable “hotspot” near the C-terminal extension by soaking ALAS2 crystals with fragments from a compound library (Fig. 4*B*). The authors identified eight fragments that bind in a hydrophobic pocket connecting helix α15 from the C-terminus to its interaction surface in the opposite subunit. A ninth fragment is bound between helix α15 and the dimer interface. Targeting interactions between the ALAS2 C-terminal extension and the catalytic core is a potential route to treat erythroid heme biosynthesis disorders. In addition, the antituberculosis drug isoniazid was found to be an ALAS2 inhibitor (69). Although clinical trials showed isoniazid to be ineffective (70), similar inhibitors may have value in treating individuals with XLP. The cocrystal structures of ALAS2 bound to this and other currently developed inhibitors may reveal new druggable regions.

The field of structural biology is primed to answer additional questions regarding how genetic mutations alter ALAS2 protein structure and activity leading to disease. No structures to date illustrate the fully formed active site with cofactor and substrates. Capturing the “active” conformation of ALAS2 will be crucial to understanding several of the disease-causing mutations. Another outstanding question in the field is how different mutations in the autoinhibitory C-terminal region cause either loss- or gain-of-function? Also, how did this region that is absent from bacterial ALAS orthologs expand to be a mutation hot spot? Next, how do structural elements not captured in the most recent crystal structure impact ALAS activity? For instance, there is a gap of structural information pertaining to a large portion of the ALAS2 N-terminal domain. In yeast ALAS, this region is known to interact with ClpX to promote PLP binding (34, 35). Although mutations in ClpX also lead to the blood disorder erythropoietic protoporphyria (71), the role of the human ALAS2-ClpX interaction is still being elucidated (63, 71). Could the structure of this variable N-terminal region reveal new interactions with other protein modulators like ClpX or with regulatory small molecules like heme, a known modulator of human ALAS1 (72)? Also, the final nine C-terminal amino acids were found to be flexible in the ALAS2 structure, opening the door for other factors to aid in stabilization. Aminolevulinic acid synthase 2 was shown to interact with two separate TCA cycle enzymes, one of which specifically binds the C-terminal extension (54, 64). Finally, there is still no experimental structure for human ALAS1, which bears ∼60% sequence identity to ALAS2; however, the N- and C-termini diverge greatly. Could the similarities and differences identified with the ALAS1 structure yield new insight into how ALAS2 functions in its erythroid niche? The recent crystallographic insight into human ALAS2 now opens the door for further structure-based investigations. Thus, future structural work targeting ALAS2 in various active conformations and bound to protein effectors will be beneficial to advance our understanding of ALAS enzymes and the molecular basis for their roles in disease.

## Conflict of interest

The authors declare that they have no conflicts of interest with the contents of this article.
